# How to Exercise Integrity in Medical Billing: Don’t Distort Prices, Don’t Free-Ride on Other Physicians

**DOI:** 10.1093/jmp/jhad043

**Published:** 2023-10-06

**Authors:** Christopher Langston

**Affiliations:** Albert Einstein College of Medicine, New York City, New York, USA

**Keywords:** gamesmanship, integrity, medical billing, professionalism

## Abstract

This paper proposes that billing gamesmanship occurs when physicians free-ride on the billing practices of other physicians. Gamesmanship is non-universalizable and does not exercise a competitive advantage; consequently, it distorts prices and allocates resources inefficiently. This explains why gamesmanship is wrong. This explanation differs from the recent proposal of Heath (2020. Ethical issues in physician billing under fee-for-service plans. J. Med. Philos. 45(1):86–104) that gamesmanship is wrong because of specific features of health care and of health insurance. These features are aggravating factors but do not explain gamesmanship’s primary wrong-making feature, which is to cause diffuse harm not traceable to any particular patient or insurer. This conclusion has important consequences for how medical schools and professional organizations encourage integrity in billing. To avoid free-riding, physicians should ask themselves, “could all physicians bill this way?” and if not, “does the patient benefit from the distinctive service I am providing under this code?” If both answers are “no,” physicians should refrain from the billing practice in question.

## I. INTRODUCTION

Heath (2020, 86) “initiate[s] a discussion about the moral dimension of physician billing” and raises several challenging cases. He characterizes billing “gamesmanship” as a violation of the integrity of professionals as fiduciaries; when physicians game the system, they act for selfish, greedy ends, rather than being fiduciaries who put client-patients’ interests first; when the entire profession promotes gamesmanship, it acts as a cartel, not a self-regulating group of experts upholding trust with client-patients. To curb this, physicians should self-regulate by encouraging integrity in billing, rather than mere compliance with documentation requirements.

Heath builds his argument within a theoretical framework that develops the functions and limitations of instrumental forms of rationality in social situations, including the conduct of professionals selling health services. This framework is generally fecund, but unfortunately, in the case of medical billing, Heath applies the wrong parts of it. The wrongness of gamesmanship is not explained by physicians’ fiduciary responsibilities or features of health insurance as a commodity; the wrongness of gamesmanship is better explained by how it distorts prices and reduces the efficiency of healthcare markets. This explanation works from within Heath’s framework but departs from his analysis about which elements to apply. I argue physicians should not free-ride on the more common billing practices of *other physicians*. Otherwise, physicians are permitted to press insurance claims to the full letter of the contract with insurers. Thus, I agree with Heath about integrity’s importance, but I disagree about how best to exercise it. This affects how the medical profession polices its members and promotes billing integrity.

## II. HEATH’S FRAMEWORK

Heath raises examples of “fiddles”—clinical, administrative, or paperwork maneuvers whose only purpose is to secure higher monetary compensation. With some plausibility, Heath proposes that trust is eroded not only when fiddles break the law’s letter by practicing billing fraud, but also when they violate its “spirit”—the “purpose or intent underlying the rules,” the clauses in insurance contracts (2020, 92).^1^ Heath assigns fiddles to the categories of *true fraud*, *honest fraud*,^2^*gamesmanship*, and *creative billing,* based on whether a fiddle obeys the letter or spirit (Table 1).

**Table 1.** Types of fiddles^3^

	Consistent with the rules	Violates the rules
Consistent with the spirit	Creative Billing	Honest Fraud
Violates the spirit	Gamesmanship	Fraud

He emphasizes the difference between *creative billing*, which is permissible, and *gamesmanship*, which violates the spirit of contractual clauses. Gamesmanship is cheating without lying.

The letter/spirit distinction is a major theme in Heath’s general approach to institutional ethics, especially business ethics.^4^ It underlies his comparisons between markets and other institutions like the law and sports,^5^ which are structured as internal competitions among adversaries that serve larger social goals: legal contests serve the goal of justice; athletic contests serve the goals of teamwork and health, among others; business contests improve product quality, lower prices, and promote overall efficiency (Heath, 2014, 100–10). In contrast, contests with no redeeming social value, like dueling or gambling, are prohibited or curtailed. In adversarial institutions, adversaries are allowed to break some conventions of everyday interpersonal morality (boxers punch each other, prosecutors attack witnesses’ character, businesses steal each other’s customers), but there are rules that constrain otherwise antisocial behavior and direct it toward the institution’s broader, prosocial purpose. The limitation of rules is that they are inherently crude and challenging to enforce, such that adversaries can one-up each other in ways that satisfy the letter of the rules but that still contradict their spirit—the larger purpose of the competition itself. Hence, there are competitive circumstances where adversaries are freed by the rules of the contest to break everyday social prohibitions, but nevertheless should exercise restraint.

With particular business practices, the letter/spirit distinction supports Heath’s contention that even when business managers are not *contractually* forbidden (by the law’s letter) from certain adversarial practices in their relationships with employees or other constituency groups within the firm, they are still *ethically* forbidden (by its spirit) because of the firm’s larger purpose, which is to instill social cohesion sheltered from naked self-interest. If managers want to take this adversarial approach with intrafirm groups like employees, then it is better to replace them with extra firm, independent contractors. Heath disparages the suggestion that managers can exploit employees as far as the letter of contracts permits as the “let them eat contracts” thesis (2014, Chapter 5). The flipside of Heath’s objection to this thesis is that managers *are* permitted to play hardball with groups *outside of the firm* such as investors, suppliers, and customers, to whom the firm is joined only by contract. Indeed, the adversarial negotiation of prices is where markets as an institution are supposed to fulfill their efficiency-maximizing purpose. I return to this important point when discussing the billing relationship between physicians and insurers.

### Heath’s Examples

Heath raises seven examples in order to illustrate the difference between gamesmanship, which breaks the rules’ spirit, and creative billing, which does not.

#### Creative billing

(A) Redescription of laparoscopic colon resection
(B) Billing a left hemicolectomy with anterior resection as an anterior resection

#### Gamesmanship

(1) Manipulating the timing of surgery
(2) Calculating obesity
(3) Scheduling follow-up care
(4) Billing liver biopsy as resection <5cm
(5) Cholecystectomy with add-on hernia repair

In creative billing cases, the billing codes enable the surgeon to describe the same surgery in different ways with different remunerative consequences. In (A), the difference comes from applying a code for encouraging surgeons to perform procedures laparoscopically (hence, less invasively, which benefits patients). In (B), Heath infers that the difference is due to an “oversight in the billing codes” (2020, 92) in which the generic description bills $18 more than the specific one, which seems superfluous. Thus, surgeons who upcode in (A) are fulfilling the rules’ spirit, while surgeons who upcode in (B) are merely not violating that spirit.

Heath’s cases of putative gamesmanship are more complicated. Only one captures unequivocal gamesmanship. Let us start with this successful example. In case (1), surgeons can bill 50%–75% more for operations that *commence* at an “unsocial hour” (10 p.m. to 7 a.m.), whereas no extra compensation applies to cases that commence during normal hours but that cross into an unsocial hour. Heath alleges that surgeons who prepone surgery until just before 7 a.m. in order to secure extra compensation are not committing fraud, but are gaming the billing system by contravening the purpose of this additional compensation. In contrast, postponing surgery until after 10 p.m. just to bill more is malpractice, because it endangers the patient and violates fiduciary responsibility. There are borderline cases where the surgeon can postpone surgery for a few hours, but still prepone it by sneaking it in before the 7 a.m. threshold in order to secure extra compensation; these borderline cases must be disambiguated according to the surgeon’s intention. Heath does not say what the spirit of the extra compensation is, but presumably, it is to credit surgeons for undertaking more acute cases to benefit patients at the expense of the surgeon’s rest, not to enable surgeons to charge a surcharge for their first case just because they arrived at work a few minutes early. Certainly, it could not be to compensate surgeons just for working longer hours, because if that were true, cases that run after 10 p.m. would also be credited. This example demonstrates how the letter and spirit come apart.

In (2), surgeons bill 25% more for obese patients, whose BMI is over 40. The purpose is to compensate for greater surgical complexity. When a patient’s weight is close to this threshold, the surgeon can weigh the patient after surgery, after they have received intravenous fluids, which may tip the measurement over the threshold. By selecting the time to weigh patients, surgeons are not misrepresenting patients’ weights, hence, are not committing fraud; but they are manipulating^6^ patients’ information for their own personal advantage, not patients’ advantage.

In (3), surgeons can schedule follow-up appointments just past the 365-day threshold so that they can bill follow-ups as new patient appointments, which pay more. However, this is not really gamesmanship, because it is inconsistent with the law’s letter: in Ontario, new patient appointments require new referrals; thus, this fiddle amounts to fraud (Heath, 2020, 93).

In (4), the surgical procedure is describable in two ways, but unlike case (B), the difference in compensation is larger (about $250), and one code is not obviously superfluous, because neither description is a subset of the other. Liver biopsies can be done via a needle (pays $87.10) or via an incision ($102.10), and the intent is purely diagnostic, not therapeutic. Typically, the target is tissue that is clearly diseased; so, including the margins of normal tissue is less useful. In contrast, an excisional biopsy is a therapeutic attempt to remove affected tissue and manifests a concern for the margins of normal tissue.^7^ Billing code S269 (“Hepatectomy—local excision of a lesion [less than 5 cm]) pays $350.65 and is part of a connected set of codes (e.g., S275, S270, S267, S271) that involve higher rates for larger resections, which are clearly therapeutic in intent. This situation in the billing codes presents a dilemma: a small, laparoscopic liver excision for purely diagnostic purposes could be billed as “biopsy via incision” or as “local excision of a lesion (less than 5cm)” (Heath, 2020, 94).^8^ It’s hard to discern the purpose behind this difference in billing codes: is it to compensate more for riskier, more invasive techniques that tend to be done in inpatient facilities? Or to compensate more for more complex therapeutic interventions with concern for margins than merely diagnostic ones? It is not clear, but the large difference in remuneration creates the strong presumption that, unlike (B), the difference between these codes is not an oversight but instead reflects some intended purpose, however, inchoate. Therefore, this case seems to straddle the line between creative billing and gamesmanship.

In (5), surgeons can bill $100 more for the repair of hernias discovered mid operation (code E764). Heath complains that this enables payment for the repair of hernias that are “clinically insignificant” (94) and “entirely incidental to the intervention and would never have provided a reason to operate” (95). Heath suggests that the surgeon who bills for such incidental hernia repairs is not violating the letter of the rules (the surgeon performed the procedure), but is violating their spirit, which, he feels, is to compensate for two independent procedures done conjointly. Like case (4), the spirit seems more indeterminate than Heath allows. A herniotomy that provides an independent reason to operate (S333) pays $222.76, which is almost twice as much as the cost of add-on hernia repair. It seems unlikely that add-on hernia repairs must be sufficient to justify surgery to merit compensation; more likely, the purpose of the billing code for add-on hernia repair is to compensate surgeons for the additional time and effort that goes into identifying and repairing these hernias, whether incidental or not. The real problem is whether to count hernias which are very small and require almost no time or effort to repair; but this is less a problem with the spirit of the rules, as a problem of vagueness in their letter: the billing codes could easily specify a threshold according to which remuneration applies only to hernias of a certain size or time to repair. Like case (3) and cases of honest fraud, the problem is not that the rules’ spirit has been violated, but that their letter is unartfully inexact.

Let us review. Case (1) is the only clear case of genuine gamesmanship, where the letter is met but the spirit violated. Cases (2) and (3) come close; like case (1), they turn on manipulating a numerical threshold, but in (2) the threshold is arbitrary and does not reflect the spirit of the rules with any precision, while in (3) crossing the threshold amounts to genuine fraud because of other safeguards in the rules’ letter. Case (4) bears some resemblance to creative billing because it turns on two overlapping descriptions, each of which satisfies the rules’ letter, but the difference in their spirit is unclear and is not an obvious consequence of oversight in the construction of the letter. Likewise, the rule’s spirit is open to interpretation in case (5): the letter is insufficiently precise to distinguish between distinct purposes. The end result is that cases of unequivocal gamesmanship are restricted to case (1).

## II. EXERCISING INTEGRITY WHEN THE SPIRIT OF THE RULES IS UNCLEAR

Our examination of these cases is consistent with Heath’s claim that there is a “moral grey zone” between fraud and creative billing (2020, 92). Heath contends that *integrity* deserves renewed emphasis in this zone, but our analysis shows that this recommendation is premature. When the rules’ spirit is unclear, it is difficult to discern genuine gamesmanship and distinguish it from creative billing; hence, integrity provides little guidance. Appeals to integrity cannot enforce uniformity on billing practices where sincere minds can disagree about the rules’ spirit. Integrity is an executive virtue, and in general, executive virtues like courage, self-control, and prudence are *amplifiers* of moral virtues and vices, while executive vices are *attenuators*. The importance of executive virtues like integrity is conspicuous only when the appropriate exercise of moral virtues and vices is clear. Thus, Heath’s appeal to integrity is hollow.

There are three ways to clarify the role of integrity. First, brighten the line between gamesmanship and creative billing. For example, require physicians to discern the spirit according to general principles of professionalism (which is roughly Heath’s position) or of business ethics (my position). Second, *maximal permission*: when the line between creative billing and gamesmanship is unclear, then physicians are permitted to press insurance claims up to the point of fraud. This proposal endorses *legalism*, the thesis that the letter of the rules exhausts moral obligations in billing. A conspicuous problem with this proposal is that it fails to generalize to other adversarial social institutions: for example, it implies rejecting sportsmanship in athletics. Third, *maximal prohibition*: when the spirit is unclear, physicians should err on the side of caution and follow integrity by avoiding the temptations of greed as far as possible. In effect, this enjoins physicians to reflexively downcode in order to avoid any appearance of greed. It renders impermissible not only in all cases of putative gamesmanship, but also of creative billing. This proposal suffers from problems of scope similar to the last. Maximal prohibition under- and over-generalizes: under because no other professions (e.g., lawyers or accountants) are subject to similar constraints in submitting claims to clients’ insurers; and over because it demands unreasonable asceticism of physicians. While maximal permission eliminates sportsmanship, maximal prohibition exaggerates it—in effect, implying that boxers should turn the other cheek. Thus, maximal permission and prohibition are unsavory extremes that depart from Heath’s framework. With this in mind, let us examine how Heath might develop the first option.

### The Argument from Fiduciary Responsibility

Suppose a physician “games” patients by inflating prices in violation of the spirit of the billing contract. Patients are not in a position to know that the prices have been inflated, because of lower medical literacy. If patients discover the physician’s gamesmanship, it will undermine trust in the medical profession and harm all physicians as a result. Therefore, the “information asymmetry” between physicians and patients generates an obligation for physicians to avoid gamesmanship. This is Heath’s Argument from Fiduciary Responsibility in a nutshell.^9^

This argument is sound, but limited: strictly speaking, it implies only a *specific* obligation to eschew gamesmanship in cases of information asymmetry, cases which never arise in common, “real world” billing contexts. Heath’s own examples illustrate how rarely information asymmetries arise: they do not arise in cases (1), (2), and (3), which all involve numerical thresholds; no special *medical* knowledge is required to understand that the patient’s weight can be measured twice and the higher number selected, or that an appointment can be scheduled just after the 365-day threshold. Physicians who game their patients in these ways could at most be accused of being price-gouging jerks who charge capricious prices, not of violating any special, *fiduciary responsibility* to patients. A patient who did not like any of these billing arrangements could easily have walked away or negotiated an agreement where the letter of the agreement operationalized the spirit more precisely. *Caveat emptor*.

A fiduciary responsibility to distinguish gamesmanship from creative billing potentially arises only in cases like (A), (B), (4), and (5), where the same medical service satisfies the letter of distinct billing codes. Luckily, this kind of situation never arises when physicians are negotiating directly with patients, because patients do not have sufficient medical knowledge to author *codes* in the first place.^10^ In the rare circumstance where patients do negotiate directly with physicians, they form a one-off contract where there is little room for slippage between the letter and spirit of the agreement. In all other cases, billing codes are an artifact of insurers, who hire *other physicians* to help craft and apply billing codes. If a physician who works for the insurer crafts a billing code and another physician submits a claim that satisfies that code’s letter but possibly not its spirit, which is unclear, then because of the information *symmetry* between the two physicians, it is false to claim that the physician submitting the claim has a special obligation to draw bright lines between gamesmanship and creative billing. Rather, the physician who is the code’s author and the insurer who employs that physician would seem to have the weight of responsibility in ensuring that there is no daylight between the code’s letter and spirit. Indeed, insurers employ physicians to oversee the application of billing codes—hence, contested claims pass through successive rounds of “peer to peer” evaluation, where the doctor submitting the claim and a doctor working for the insurer discuss the details of the case.^11^*Such processes eliminate the information asymmetry between the billing party and the billed party*, or at least substantially attenuate it; *where there is no information asymmetry, there can be no special obligation not to exploit it. As such*, fiduciary responsibility is irrelevant.

Heath may have overemphasized fiduciary responsibility in particular^12^ as the most prominent of several duties of professionalism (2020, 99), which are intended to promote trust and extend beyond information asymmetries alone. For example, physicians can collectively agree that felonies like arson should be professionally disqualifying, even though the wrongness of arson does not turn on an information asymmetry, or even on fiduciary responsibility more generally; arson is disqualifying because having felons among the professional ranks would reduce patient trust in the medical profession. Similarly, general duties of professionalism may support prohibitions on billing gamesmanship, even when gamesmanship itself does not turn on information asymmetry. Insofar as Heath alleges that gamesmanship exists in a “moral grey zone” between fraud and creative billing, he seems to concede that one cannot expect the gamesmanship/creative-billing distinction to be enforced strictly; rather, professionalism enforces it loosely through integrity-based culture (2020, 98–9). This culture prevents mischief in the grey zone but does not necessarily ensure uniformity of practice.

There are two overlapping problems with a professionalism amendment to Heath’s primary argument. First, it lacks defined scope and so threatens to under- and over-generalize about billing restrictions in the same ways as the maximal prohibition proposal, thereby conflicting with Heath’s framework. For instance, when Heath says that professionalism enjoins physicians to “refrain from acting opportunistically, thereby creating trust” (2020, 98), the limits of “opportunistic” action are undefined. It satisfies one sense of “opportunistic” for physicians to charge capricious prices and offer “tiered” care (concierge medicine, VIP floors in hospitals, “Medicaid clinics,” etc.), but typically these have no appreciable effect on patient trust. Also, opportunism should not generate distrust if it is part of general business dealings; any professional prohibition on opportunism cannot be so strong that it turns doctors into suckers, but it’s unclear how professionalism as such could formulate a prohibition of the right strength and scope. Consider a patient who overhears the physician on the telephone, but it is unclear whether the physician is pressing a health insurance claim on the *patient’s* behalf, pressing the same claim for *the physician’s own* gain, or pressing a property insurance claim for the physician’s own gain because of water damage in the clinic’s bathroom. The patient, the patient’s insurer, and the physician’s property insurer are all external contractors of the physician’s business venture. The professionalism amendment aims to reach the conclusion that patient trust would not be affected by physician greed as such (i.e., not for high concierge prices, or for the physician’s own gain against the property insurer), nor by gamesmanship as such (i.e., not against the patient’s insurer for the patient’s gain), but would be affected only in the combined case when the physician is pressing a claim for the physician’s own gain *against the patient’s insurer*. It is hard to see how professionalism as such strikes this delicate balance; it is unlikely that the patient overhearing the doctor on the phone would be listening attentively for such subtle details in order to condemn or absolve the physician. Rather than strike this right balance, appealing to professionalism would seem to smuggle interpersonal morality into adversarial institutions through the back door: rather than tell boxers to avoid blows to the back of the head, it would tell them “punch softly”; and rather than tell football players to avoid unnecessary roughness, it would tell them “tackle gently.” The prescriptions are close, but insufficiently precise and with the wrong emphasis.

Second, it is an *empirical* question as to what extent billing gamesmanship affects patient trust.^13^ Indeed, it is highly dubious that patient trust is *generally* undermined by gamesmanship, especially in cases where physicians violate the billing code’s spirit *in order to benefit the patient*. More generally, patient trust is affected by how gamesmanship is framed in terms of the wider context. When characterizing this context, Heath paints in broad strokes: he characterizes physicians who practice gamesmanship as greedy cheaters who exploit the Ontario Health Insurance Program (OHIP), the people’s champion. He contends physicians rationalize sharp billing practices with sanitizing language; they amplify grievances over their compensation, which he portrays as ample; and they practice what psychologists call “moral disengagement,”^14^ against which integrity is a bulwark. These points are less developed and more controversial than he acknowledges. Under this tendentious characterization, it is easy to see how patient trust could be jeopardized. Of course, its tendentiousness is why Heath does not lean heavily on it; instead, he raises his second argument for why physicians have a special obligation to distinguish gamesmanship from creative billing. This argument serves two roles: it adds color to the wider context, and it highlights independent reasons to sharply distinguish gamesmanship from creative billing.

### The Argument from the Susceptibility of Insurers to Inflated Claims

Heath contends that anyone filing an insurance claim has a distinctive obligation not to exaggerate or inflate the claim (2020, 97). He observes that insurers are especially susceptible to inflated claims because the extra cost is divided between all of the premium payers, and humans are psychologically predisposed to downplay harms when they are diffused. Heath urges that the temptation to downplay diffuse harms should be resisted, and this resistance requires exercising integrity. Heath recommends that claimants test their integrity by asking whether they would pursue the inflated claim if it were passed on to a single individual, instead of diffused across all of the premium payers. When physicians file a claim with an insurer, they should ask themselves whether they would be comfortable justifying the inflation of the claim to the individual patient—in other words, since the insurer is the proxy of patient interests, the effects of diffusing harm can be filtered out by substituting a single patient for the insurer. In (1), physicians who start surgery at 6:59 a.m. in order to bill more would find themselves unable to justify this practice to a singular patient and so would recognize that the practice is wrong.

There are two parts to this argument: the observation that insurers are susceptible to inflated claims; and a test for identifying when a claim has exploited this susceptibility. The first part does not *show* that inflating claims is wrong; it just shows that *when* the insurer has been harmed, this is less likely to be noticed than when a single patient has been harmed. Heath develops this part by discussing fraud and “white collar crime,” and then tries to generalize the point to any inflated claim, including gamesmanship. He insinuates that the same considerations that support a distinctive obligation to exercise vigilance in avoiding fraud support an obligation to avoid gamesmanship, but he never identifies these considerations: fraud is wrong because it is *theft by pretense*, but by definition, gamesmanship contains no pretense, hence, no fraud. This part of the argument succeeds only if the wrong-making feature of gamesmanship is supplied from elsewhere, such as the argument from fiduciary responsibility, or the second part of the susceptibility argument, the single-patient substitution test. In the last section, we defused the former. Here, we show that the latter fails because insurers are not proxies of patients’ interests.

Heath’s substitution test is problematic in principle (even after we put aside the practical dissimilarities between patients and insurers^15^). In order to screen out any diffusion of harm, Heath’s test turns on switching from the indirect billing context, where the insurer is billed, to the direct billing context, where the individual patient is billed. This test works in the case of fraud since the same grounds of harm hold in both the direct and indirect billing contexts. Now in the case of gamesmanship, the transition from one context to another removes not only the diffusion of harm, but also the grounds of harm in the first place. When a surgeon is in a price negotiation with a single patient, the surgeon can request a surcharge for cases begun during unsocial hours; if the patient does not want to pay the surcharge, then the patient can ask for a cheaper surgery time or simply find another surgeon who does not charge this surcharge. If the patient agrees to pay it, *then no wrong has been committed*. In the direct billing scenario, the surgeon is only guilty of setting capricious prices, which is the surgeon’s prerogative in the medical marketplace; the surgeon has not obviously harmed or wronged the patient, especially by being unprofessional.^16^ So, even if physician gamesmanship harms the *insurer*, where this goes unnoticed because of the diffusion of harm, it does not help to transpose the same practice to the direct billing context, where the *patient* is *not* harmed—hence no wrong committed.

The direct scenario described above might seem like an outlier because typically patients cannot shop for surgeons. Except with elective surgeries, surgeries are urgent/emergent cases that come via ambulance to the emergency department without any patient choice in the matter. In these more typical contexts, it might seem ethically problematic for a surgeon to request a surcharge after preponing a patient’s case from 7:15 to 6:45 (recall that the surgeon cannot *postpone* the case solely to increase payment without committing malpractice): the patient seems to have no ability to bargain. There are at least two problems with this objection. First, emergency situations prompt generalizations to the worst case; so, they should not be used as a counterexample that health services are traded in a true market (compare this argument with the similar argument that flooded basements are not good grounds to argue that there is no true market for plumbing services). The more general phenomenon is that the demand for health care is very price elastic in the long run (patients defer cheap health maintenance) and very inelastic in the short run (patients want their current medical problems solved immediately regardless of cost); emergency situations are a limiting case. Second, even when the patient cannot shop around between surgeons, the patient can still bargain: if the surgery is being preponed for a surcharge, the patient can make the decision whether it is worth saving the additional money to delay surgery—a delay, which, by hypothesis, is not medically significant because otherwise the surgeon would have been prohibited from offering it. So, no matter how it is specified, the patient is not harmed or wronged in the direct scenario. Because no harm/wrong has been done to the patient in the direct billing scenario, no harm/wrong has been done to the insurer in the indirect scenario either—the insurer expresses the same preference for earlier surgery, but on a larger scale.

### Disentangling the Arguments

Now we are in a position to disentangle Heath’s two arguments. These arguments turn on two asymmetries between physicians and the parties they are billing. When these asymmetries are teased apart, it becomes clear that each asymmetry on its own is insufficient to establish an obligation to eschew gamesmanship and that putting them together does not gain any additional traction. The Argument from the Susceptibility of Insurers to Inflated Claims aims to generate a responsibility to eschew the gamesmanship of *insurers*, and the Argument from Fiduciary Responsibility (with its professionalism amendment) aims to generate a responsibility to eschew the gamesmanship of *patients*. The substitution test tries to connect them by transferring physician’s fiduciary/professional responsibility from being directed toward patients to being directed toward insurers; conversely, the susceptibility of insurers to inflated claims affects the wider context in which patient trust is formed and in which physicians exercise their professional responsibility to patients. Thus, the two arguments have interlocking features; in conjunction, they form a larger argument for physicians’ responsibility to root out gamesmanship. When this is characterized as physicians greedily exploiting nonprofit OHIP, then the wrongness of gamesmanship seems not just obvious, but so egregious that integrity deserves renewed emphasis.

The analysis presented here shows why the two arguments must be separated: the fiduciary responsibility that physicians have to patients does not apply in almost any practically relevant billing contexts, and even then, it is not absolute; it must be considered alongside other factors affecting patient trust. The professionalism amendment extends these duties to prohibit gamesmanship, but at the cost of indeterminacy and unclear scope that threatens to conflict with Heath’s framework. The susceptibility of insurers to inflated claims generates an obligation to vigilantly avoid fraud, violations of insurance contracts’ *letter*. However, without the argument from fiduciary responsibility, it does not extend to gamesmanship, violations of their *spirit*. Moreover, it is fallacious to treat insurers as proxies of patient interests such that obligations to patients carry over to insurers. Each argument on its own is insufficient to show that billing gamesmanship is wrong, let alone that physicians have a responsibility to detect and avoid it. Accordingly, the emphasis on integrity is premature.

## III. WHEN AND WHY GAMESMANSHIP IS WRONG: THE PRIMARY-FUNCTION-OF-PRICES ARGUMENT

I borrow from Heath’s framework the insight that gamesmanship is wrong because it violates the spirit of insurance contracts. Where Heath construes spirit in terms of fiduciary/professional responsibility, I construe it more broadly as markets’ primary function, which is to carry information about where resources are needed, and more narrowly as undistorted pricing. By price “distortion,” I mean that a physician submits insurance claims to intentionally exploit a loophole that is not universally available to other physicians (hence “free-rides” on standard practice) and which the physician believes does not represent an improvement in service compared with less remunerative codes. In short, it is unequal pricing that fails to produce a pareto improvement. When successful, this attempt to exercise market power produces a deadweight loss that foils the purpose of the market. Compared with Heath’s proposal, mine is much more permissive.

Let us work up to this conclusion by reexamining Heath’s cases—which cases are ethically problematic and why? Case (1) seems the most problematic, even though the physician does not take advantage of an information asymmetry, violate patient trust, or flagrantly exploit the susceptibility of insurers to inflated claims. There are indications that this practice is ethically wrong: the physician who schedules early surgery for extra compensation acts unseemly; physicians would hardly be proud of this practice (indicating “consciousness of guilt”). I hypothesize that the gamesmanship in (1) is wrong because it is not correctible in principle, hence distorts the prices of a medical service. This transmits the wrong information to the market and produces a deadweight loss. The result is that the function of the healthcare market is foiled.

Health care is a commodity, but it is not traded in a classical market. Patients bundle their demand for health care and pay for health services through insurers. Bundling is more efficient than saving for health care individually, because economy of scale reduces the variance in each individual’s expected healthcare expenditures. Currently, the insurance market is dominated by publicly administered companies, like OHIP and Medicare, which enjoy a (near-)monopoly over health *insurance*, and a (near-)monopsony for health *care*. In this respect, they effectively set prices and resemble “buyer cartels.”^17^ Despite the intricacies of the quasi-market for health care, its price structure still displays important features of prototypical markets.

The main function of prices is to carry information about the demand to suppliers and about supply to consumers: when prices go up, the market effectively says, “produce more” and “consume less”; when prices go down, it says, “produce less” and “consume more.” This is true even in the quasi-market for health care,^18^ but it can be obscured by the interposition of insurers between the physician (the supplier) and the patient (the end consumer). Suppose that when insurers like OHIP or CMS update their fee schedules, they increase payments for 10 p.m. to 7 a.m. surgery; this tells surgeons, “produce more 10 p.m. to 7 a.m. surgery,” and tells patients and their insurers, “consume less.” However, the demand for such surgery *among insured patients* does not change *directly* because they are shielded from prices. Instead, their insurers, who are exposed to the price increase, pass it along to patients *indirectly* in the form of increased premiums, co-pays, or deductibles (or taxes, with public insurers); or insurers fiddle with the “effective price” (i.e., limiting consumption on patients’ behalf) by adding various restrictions on supply, such as through “step algorithms.” In the limit, insurers drop coverage for certain kinds of care, in which case patients become uninsured. Uninsured patients limit their own consumption as in classical markets. Although the price mechanism in health care is different from in classical markets, it still has the same primary function, just with clunkier execution.^19^

Gamesmanship like that in case (1) produces *noise* in the price information stream. The fiddle in case (1) is not universalizable; it is possible only in small surgical centers where individual surgeons have discretion over work hours; at large institutions, where nursing and staff work schedules are tightly regulated, surgeons do not have the unilateral authority to perform this fiddle, and if this practice were adopted system-wide at such institutions, insurers would easily take notice and restrict the code to explicitly acute cases (e.g., claims for surgeries during unsocial hours might require additional documentation stating that delay posed an urgent danger). This kind of correction is what happens in case (2), where insurers require new referrals for new patient visits, because insurers realize that it is possible to schedule appointments just past the 365-day threshold. The corresponding correction is practically impossible in case (1). Re-organizing a large hospital’s surgery schedule is like asking, “please move the hospital a little to the left.” After extensive negotiations with nursing, pharmacy, anesthesia, etc., over many months, the change could be implemented, but it would be foreseeably futile since insurers would foil it easily. It would be a massive but ultimately pointless undertaking. In contrast, small surgical centers can fly under insurers’ radar.^20^ There may be borderline cases of “near-universal” or “almost correctible” billing practices that require special treatment, but case (1) does not seem like one of them. Consequently, (1) is a case of parasitic free-riding on other physicians’ billing practices. When small surgical practices capture the payment bump for 6:45 a.m. surgery, their income increases until a new equilibrium with surgical demand is reached. From here, the story takes one of two turns, which are indistinguishable from society’s point of view: either society tolerates this increase and shifts production away from other sectors to surgery; or it does not, in which case, insurers indiscriminately slash reimbursement for surgery. If there were palpable benefits to pre-7 a.m. surgery (e.g., patients could return to work earlier), then this shift in production might be worth it, but in case (1) patients do not benefit. Gamesmanship draws more resources to health care without any consumer benefit; compared with the market-clearing price, this exercise of market power yields a deadweight loss.

Now, even if I am wrong about the facts of case (1), this error would not undermine universalizability as a prohibition criterion. Ultimately, gamesmanship turns on the physician’s state of mind: if any physician sincerely believes that the maxim of his action is universalizable, then the act does not amount to gamesmanship. In contrast to gamesmanship, “creative billing” consists of fiddles that are licit because the physician believes they either represent an improvement in product quality or are universalizable, hence, correctible, and do not distort prices. Consider case (A). When new techniques like laparoscopy^21^ are first introduced, they are usually available only at large academic centers; the new technique is diffused through the rest of the surgical community. During the transition, some centers can perform the new procedure and apply the more remunerative billing code, while others cannot. But the surgical techniques and their associated billing codes are not impossible to universalize in principle; the limitation is only temporary. Moreover, even if this differential existed in principle (which might be the case with services like proton beam therapy for cancer, for example), it would still be justified because it benefits patients. This is just the exercise of competitive advantage.

Even when a billing practice does not benefit patients, it can still be licit because it is universalizable, hence, correctible. Consider case (B): even though patients do not benefit when the same procedure is billed as “anterior resection” instead of “left hemicolectomy with anterior resection,” this difference is universalizable; all physicians could apply the generic code instead of the specific one. When universally adopted, insurers are able to assess whether they are paying too much for the generic code, and simply correct the amount they are willing to pay, in which case the price might return to what it would be had everyone billed for the lesser code. When services are billed *uniformly*, the market bears the same price for the same service. That is, even though the difference in case (B) does not represent any improvement in quality, it is universally possible to adopt the higher billing code, and the resulting uniformity allows the price to return to the right equilibrium. Similarly, in cases (3), (4), and (5), if all physicians applied the more remunerative code, then insurers could assess whether they were paying more than they were willing to for these services and adjust payments accordingly. Therefore, these cases are not ethically problematic. They provoke suspicion of “gamesmanship” only because of the opportunity to apply the codes nonuniformly, but this suspicion does not survive scrutiny.

The preceding argument presupposes that insurers can adjust prices flexibly, which is not necessarily the case. For example, OHIP updates its schedule every four years; CMS annually. With such delays, there is a perpetual gap for mischief, a gap which insurers play catch-up to close. Excusing upcoding during such periods might seem similar to excusing pollution when the government has not (yet) outlawed it. However, such worries should not be overblown. Annual updates are reasonable for prices affecting 10%–20% of GDP; more frequent updates might have unforeseen but problematic consequences. Furthermore, insurers are not necessarily playing catch-up; they have ample opportunity to anticipate trends. Poor business management (e.g., delayed updates) is never a reason to demand generosity in an adversarial contractual negotiation.

On my hypothesis, when physicians are seeking to detect and avoid gamesmanship, they should ask themselves, “could all other physicians bill the way I am billing?” and, if not, “does the more remunerative code reflect a genuine competitive advantage?” If the answer to both questions is “no,” then they should refrain from the practice. This set of questions comprise a test that is different from Heath’s substitution test, in which physicians ask themselves, “would I bill this individual patient the same way I am billing the insurer?” There is some overlap between the two tests, insofar as it would pass both tests for physicians to apply a more remunerative code that reflects a benefit to patients (e.g., case [A]), or not (e.g., case [1]). But in muddy cases like (B), (3), (4), and (5), Heath’s test is less reliable; it requires physicians to indirectly test their own motives by speculating about the reactions of patients, who are ignorant of billing codes and the billing process. In contrast, my test requires physicians to ask themselves whether they believe potential nonuniformities in billing are due to a genuine improvement in service. Not only is this test easier for physicians to apply, but also it discriminates which practices are licit more decisively. Heath’s test produces uncertain results for cases (B), (3), (4), and (5), whereas my test delivers the clear verdict that physicians are permitted to bill aggressively.

So long as they do not distort prices, physicians are permitted to press billing claims, just as insurers can resist them in return. This is one of the important lessons of Heath’s framework that we developed in Section I outside of the firm, the rigorous and exacting enforcement of the letter of contracts enables markets to function efficiently. The spirit of contracts matters too, but the relevant aspect of “spirit” concerns the possibility of uniform pricing. Restraint is called for when physicians can flout the spirit of billing codes in ways that distort prices—in the first instance, non-universalizable ways that do not reflect a benefit to patients.

In my analysis, gamesmanship is wrong because physicians free-ride on other physicians, not because they are cheating insurers or patients. Gamesmanship is not necessarily against physicians’ collective self-interest. Physician A can adopt gamesmanship practice alpha to free-ride on the billing practices of physician B, who can in turn adopt gamesmanship practice beta to free-ride on the billing practices of A. Each physician benefits personally, and physicians benefit overall. So free-riding need not result in overall harm to physicians. But the gamesmanship is still wrong, because it distorts prices in the healthcare market. The vice at issue in gamesmanship is *cheating society without lying*; integrity is a bulwark against this vice, but the way integrity must be exercised is different than if the relevant vices were exploitation, lying, stealing, breaking promises, or other ways of contravening fiduciary responsibilities.

My analysis explains what Heath’s analysis gets right and wrong. The *Cui Bono* (who is benefitted?) of gamesmanship is clear: physicians. But the *Cui Malo* (who is harmed?) is not. Heath tries to tie the harm to the insurer (via the Argument from the Susceptibility of Inflated Claims) and to the patient (via fiduciary responsibility, or professionalism). The problem is that the insurer, who is directly harmed, has no basis for complaint, since the insurer wrote the rules by which the physician is billing; and the patient, who is indirectly harmed via increased prices/premiums, has no control over the billing codes. Consequently, gamesmanship is not wrong because it is a violation of these “first-order” duties to insurers and patients. The more compelling part of Heath’s analysis is when he suggests that gamesmanship is wrong because of how it affects the *generic* patient, as the payer of premiums, and OHIP, as the insurance market writ large. The insight here is not that individual patients or OHIP are harmed in an indirect way; the insight is that gamesmanship impairs the function of the healthcare market within the entire society; these shifts in production harm society’s nonhealthcare projects.

Once the wrong-making feature of gamesmanship has been successfully identified, only then does the susceptibility of insurers to inflated claims matter: when many small price distortions are diffused among premium payers through their premiums, it is harder to identify the harm, and the wrongness of gamesmanship *qua* cheating is easy to overlook. Now when physicians cheat billing codes in ways that increase premiums, this can have an indirect effect on patient trust. Neither of these effects of gamesmanship is sufficient to explain why gamesmanship is wrong on its own, but both are aggravating factors that make it *worse*.

My analysis provides guidance for professional schools and organizations teaching and policing their members. Gamesmanship is wrong because it foils the function of the healthcare market. Therefore, the profession as a whole has an interest in squashing it. When physicians share advice on billing tips and tricks to maximize payment, or when professional organizations host seminars to avoid “leaving money on the table,” these educational activities induce billing *uniformity* and reduce opportunities for free-riding. This uniformity aspires to maximize compensation, which can cross into crass opportunism, but it also has the benign purpose of educating physicians and correcting their tendency to underbill. Many physicians struggle to recoup full payment for their work. Not only do they fail to submit billing for some cases, but also a substantial portion of their bills is denied; insurers “claw back” payments years later for reasons that are difficult for physicians to rebut so long after the original service. In this cutthroat billing environment, it makes sense for physicians to promulgate billing advice widely and to encourage uniformly maximal claims. While preparing future physicians for this environment, medical schools should teach students the tests for identifying gamesmanship proposed here.

## IV. CONCLUSION

Physicians sell services to patients, who pay their bills via insurers; physicians maintain billing contracts with these insurers and submit claims to them for services provided to patients. Physicians have distinct duties to their patients and the insurers with whom they contract. They also have duties to their colleagues and to the well functioning of the profession in the healthcare marketplace. It is important to disentangle which duties physicians have to which parties. The purpose of this convoluted payment system is no different from any other market: to direct resources to where they are most needed. Billing gamesmanship violates the spirit of this institution; it is a form of legal cheating that distorts the price mechanism and interferes with the point of this market. The best way for physicians to detect gamesmanship is to ask themselves, “Could other physicians bill the same way that I am billing?” If the answer is “no,” then they should ask themselves, “Does this way of billing benefit patients and exercise a competitive advantage against other physicians?” If the answer to the second question is also “no,” then they should refrain from the practice. Otherwise, physicians are permitted to press their claims against insurers to the maximum extent. Physicians and their professional organizations should encourage uniformity in billing practices in order to minimize price distortions, and they should reinforce the importance of integrity in avoiding gamesmanship. These duties are not unique to *medical* or even *professional ethics*; they are just good business practice.

## Footnotes

1 Gamesmanship involves *intentional* malfeasance, which is different from *abuse*, which involves *unintentionally* erroneous billing that violates billing codes’ letter. Cf., the American Medical Association’s (2017)*Principles of CPT Coding, 9*^*th*^*Ed.*

2 Heath does not elaborate on honest fraud because it is a species of fraud; he includes it in his taxonomy to illustrate the letter/spirit distinction. He insinuates that honest fraud is less seriously wrong than true fraud because it reflects “a problem with the rules, such that when applied literally they do not provide fair compensation” (2020, 93).

3 Reproduced from (Heath, 2020: Table 1).

4 Cf., Heath (2014), Chapter 4.

5 Cf., Heath (2019).

6 Heath is not completely convinced that this is gamesmanship. He acknowledges that the difference in surgical difficulty between a BMI of 39 and 41 is negligible and that 40 is an arbitrary threshold. He proposes a graduated billing scheme might operationalize the spirit of this compensation regime better. The problem is like that of honest fraud (cf., footnote 2), the difference being that in this case there is no deception, hence no violation of the letter.

7 One surgical guideline notes that excisional biopsy is “often used for biopsying lesions found fortuitously at routine laparoscopic surgery,” though it adds, “some centres in the USA perform laparoscopic liver biopsy on outpatient basis” (Grant and Neuberger, 1999, IV2), where this suggests the biopsies are preplanned, not fortuitous.

8 Heath says that upcoding for a diagnostic liver resection “is obviously right on the border between gaming and fraud” (2020, 94), but this characterization seems mistaken: it presumes that the spirit of the rules has “obviously” been violated, and the open question is whether the letter has been broken, too. But actually, the situation seems to be the reverse: small excisions clearly satisfy the letter of the more remunerative billing code; its spirit is unclear.

9 The argument is spread over section IV of Heath (2020), but is concentrated in the last full paragraph on page 100: “[I]t is easy to see what the problem with the current attitudes toward billing [is] in the medical profession. Just as in the case of patient care, the fundamental issue involves an information asymmetry.”

10 Indeed, the patient’s comparative medical illiteracy is one of the critical reasons that physicians submit claims to insurers directly, rather than having patients seek reimbursement.

11 There is another feature of these cases that underscores that physicians do not enjoy an informational advantage. In many practices, physicians do not submit claims themselves, but hire practice managers as professional coders to review clinical documentation and submit the most remunerative set of codes. Despite their knowledge of billing codes, practice managers do not have more *medical* knowledge than physicians—the knowledge relevant to exercising fiduciary responsibility. Hence, compared with physicians employed by insurers, practice managers are at an informational *disadvantage*; if there is any asymmetry in medical information, it would be in *the insurer’s favor*. Moreover, practice managers are deputized agents of physicians, while insurers are deputized agents of patients. As Heath argues elsewhere (2014, Chapter 10, esp. 247), agents are bound by all of the principal’s obligations and prohibitions (although agents can have additional obligations and prohibitions that are only permissions of the principal). If physicians exercise fiduciary responsibility in medical billing, then, as their agents, practice managers would, too. This would fly in the face of current practice, where practice managers show limited concern for patients’ interests (cf., AHIMA Standard of Ethical Coding, 2016). Current practice may be wrong, but I submit that the more natural interpretation of current practice is that because the fiduciary relationship is irrelevant in the direct billing scenario between physician and patient, it is not carried over to the doubly indirect billing scenarios between practice managers and insurers.

12 As previously mentioned, Heath says, “the *fundamental issue* involves an information asymmetry” (emphasis added). To my ear, “fundamental” gives the distinct impression that he thinks the wrongness of gamesmanship turns on an information asymmetry, though he also discusses general duties of professionalism (Heath, 2020, 98–9).

13 The reaction of patients is relevant because it reflects genuine trustworthiness, which in turn establishes limits for professional duties. Thus, I am *not* claiming that doctors are permitted to practice gamesmanship so long as they “get away with it”; I am merely claiming that patients’ reactions serve as a barometer of trustworthiness in this case.

14 Cf., Bandura (2002).

15 On the practical level, insurers are not proxies of patient interests because insurers have their own agenda, especially for profit insurers, who serve their bottom line, not patient interests. Physicians enter into contracts with commercial insurers that are negotiated within the marketplace, and physicians are permitted to “play hardball” by holding insurers to the letter of these contracts, just as any other business does, such as when Wal-Mart levies fines against vendors who fail to satisfy contracts precisely (Gerstein, 2017). Any dollar that a physician “leaves on the table” goes into the insurer’s pocket, not back into the patient’s. In practice, physicians have limited ability to play hardball with commercial insurers when it comes to recouping claims. American physicians regularly and intentionally “overbill” on claims that they submit to commercial insurers, because they have no idea what proportion of the charge the insurer will actually pay; if the physician charges less than what the insurer actually pays, then the physician “leaves money on the table” and incurs a self-inflicted loss. (This is described with concrete examples in Chapter 5 of Belk and Belk, 2020.) As described in Section I, Heath’s approach to business ethics implies that hardball billing practices with insurers are permissible because markets achieve their efficiency-maximizing function through such self-interested price negotiations. There is no difference when physicians bill aggressively according to the letter of contracts, than when one boxer punches another in the face—this is part of the adversarial institution. The suggestion that doctors “play nice” with insurers thus risks committing an error that Heath objects to elsewhere, viz. it is fallacious to transplant the norms of “everyday” morality into institutional contexts that are self-consciously adversarial. So, in both the formation of contracts and their performance, physicians interact with insurers in ways unlike the way they interact with patients. It is inappropriate to treat insurers as proxies of patients; the substitution test is not apt. These practical problems with Heath’s test are obscured by his test case: physicians in Ontario, Canada, who do not negotiate prices with commercial insurers but rather accept a price schedule from the Ontario Health Insurance Program (OHIP), which is nonprofit and publicly administered. (In this respect, Heath’s insistence [2020, 87–8] that the situation of these physicians generalizes to physicians at large is strained). But even nonprofit, publicly administered health insurers like OHIP or Medicare are not straightforward proxies of patient interests. As Heath points out elsewhere (2003, 2014, Chapter 2, section 2.4 “Lessons from Public Management”), public insurers are not charities; they sell a commodity and have governance structure like commercial firms. To the extent they are enmeshed in the political bureaucracy, they serve multiple stakeholders, such as politicians, academic medicine, research consortiums, physician groups, pharmaceutical companies, and private insurers. They are a funny mirror reflection of patient interests, not a window onto them.

16 To help see this, imagine an inverted scenario, where a surgeon bills the “regular price” from 10 p.m. to 7 a.m., but offers a “discount” from 7 a.m. to 10 p.m.. A patient is offered the “regular price” at 6:59 might be disappointed that they missed out on the “discount,” but they have not been wronged or harmed because they missed it.

17 I stress this point because Heath compares professional organizations to seller cartels, but does not mention the demand side mirror image of buyer cartels.

18
Frakt and Chernew (2018, E1) observe: “Prices are signals, and even in health care, many individuals follow them in some circumstances. The problem in health care is not that prices play a role – that is unavoidable. The problem is that prices are distorted in ways that result in an inefficient allocation of healthcare resources.”

19 There are many complications with this story: public-private subsidization and “cost-shifting”; “queue jumping” and crossed financial streams; connections between Canadian and US health care (e.g., OHIP patients getting “buffaloed”); vertical integration between physicians and insurers (e.g., health maintenance organizations) or between physicians (e.g., accountable care organizations), which can resemble “seller-cartels.”

20 Another possibility: large surgical centers might consider taking a “if you can’t beat ‘em, join ‘em” approach and distribute some of their case load to smaller centers who could adopt this practice and with whom they could form partnerships. But, the profits from such ventures would likely either be too low to offset the administrative efforts or would again attract attention from insurers.

21 This is a fictionalized version of the history of laparoscopy, which evolved over many years. Cf. Kelley (2008).
